# Quality evaluation of living and postmortem Chinese mitten crabs (*Eriocheir sinensis*)

**DOI:** 10.1002/fsn3.1519

**Published:** 2020-04-17

**Authors:** Yahui Wang, Yaozhou Zhu, Wenzheng Shi, Xichang Wang

**Affiliations:** ^1^ College of Food Science and Technology Shanghai Engineering Research Center of Aquatic Product Processing and Preservation Shanghai Ocean University Shanghai China; ^2^ Shanghai Totole Food Col, Ltd Shanghai China; ^3^ Gruma Corporation (Mission Foods) Irving TX USA

**Keywords:** chemical analysis, Chinese Mitten Crab (*Eriocheir sinensis*), flavor, putrefaction, quality assurance

## Abstract

Total volatile basic nitrogen (TVBN), biogenic amine, total viable count (TVC), volatile compounds, and sensory evaluation were conducted to assess the quality of Chinese Mitten Crabs (*Eriocheir sinensis*) at living, zero, 2, 5, 10, 15, and 24 hr postmortem. The sensory evaluation found a noticeable odor of spoilage becoming evident 10 hr postmortem. The TVBN value increased and then decreased as time increased, reaching 23.67 mg N/100 g at 24 hr postmortem. Although biogenic amines were detected at 5 hr postmortem, by 24 hr postmortem these had not reached dangerous levels of toxicity. The initial TVC (6.06 Log CFU/g) of the living crab samples was relatively high and climbed further postmortem, reaching 10.00 Log CFU/g 24 hr postmortem. Trimethylamine was detected in the living sample in belly meat and 2 hr postmortem in crab roe and reached 8.33 µg/g in the roe 24 hr postmortem. Indole was detected at 0 hr (belly meat) and 10 hr (crab roe) postmortem, but did not change significantly during the observation period. Sulfur‐containing compounds were detected 5 hr after death and gradually increased over the observation period. Most indicators showed major changes at 5 hr and 10 hr postmortem. By 10 hr postmortem, the crab had entered the putrefaction stage and was thus no longer safe for consumption.

## Graphical abstract

This is the first research on the quality of Chinese Mitten crab at living and postmortem stage to the best of our knowledge. Quality indicators for the crab of early postmortem were investigated. A safe consumption timeline for Chinese Mitten crab has been suggested.

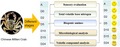

## 1 INTRODUCTION

Chinese mitten crab (*Eriocheir sinensis*) is a traditional food popular in China and has been spread as an invasive species and found in Europe and North America as well. It has a unique, pleasant aroma, and delicate flavor. According to the China Fishery Yearbook (FBMA, 2017), the annual production of Chinese mitten crab reached 812,103 tons in 2016. Due to its high economic benefits (sold at $30–50/kg), the aquaculture of *E. sinensi*s is becoming common in the fishery industry and it is now one of the most popular commercial crustacean products in many parts of the country. However, compared to fish, Chinese mitten crab is highly perishable due to its relatively high levels of free amino acids, nitrogenous compounds, and microbes (Anacleto et al., 2011; Jay, Loessner, & Golden, 2005). *E. sinensi*s is also a seasonal product whose survival time is limited within farming environments. This places substantial time pressure on both the farmers producing the fresh product and those marketing it to consumers.

Previous studies have generally focused on the quantification of the sensory score, K values, total volatile basic nitrogen (TVBN), biogenic amines, and total viable count (TVC) of bacteria and volatile compounds (Huidobro, López‐Caballero, & Mendes, 2002; Oguri, Enami, & Soga, 2007; Shalaby, 1996; Snellings, Takenaka, Kim‐Hayes, & Miller, 2003). Research on the sensory evaluation of crab meat has tended to examine the effect of the crab meat's sensory score on the product's shelf life (Lorentzen, Skuland, Sone, Johansen, & Rotabakk, 2014; Sarnoski, O’Keefe, Jahncke, Mallikarjunan, & Flick, 2010); few have looked at how these sensory scores change with time once the animal has died.

Large amounts of biogenic amines, especially histamine, putrescine, cadaverine, and tyramine, can cause allergy‐like symptoms and food poisoning after ingestion (Hwang, Chang, Shiau, & Cheng, 1995); volatile compounds associated with the corruption of crab meat mainly consist of trimethylamine (TMA), indole, and S‐containing compounds. Solid‐phase microextraction (SPME) is considered an effective way to test for volatiles in food as it can be used to extract compounds that contribute to odor or spoilage without altering their structure (Majcher & Jeleń, 2009). A gas chromatography–mass spectrometry (GC–MS) system can then be used to separate, identify, and semi‐quantify the volatile compounds extracted and has thus been widely applied to crab odor analysis (Gu, Wang, Tao, & Wu, 2013).

There have been studies on the influence of various treatments on Chinese mitten crab shelf life and quality, but few on the temporal characteristics of spoilage in untreated crab. As the traditional Chinese folk wisdom forbids eating crab that has been dead overnight, our objective was to establish characteristic indexes for the quality of Chinese mitten crab from a living state to the early stages postmortem. The current study examines what happens in crab samples that have died naturally, focusing on how five aspects of crab quality are affected by length of time postmortem, namely processing characteristics, sensory, physical, chemical, and microbiological factors. We hope that our results will provide a useful theoretical basis for improving the quality and safety of mitten crab products for human consumption.

## 2 MATERIALS AND METHODS

### 2.1 Sample preparation and processing procedure

A total of 60 female Chinese mitten crabs (average initial weight 137.79 ± 14.62 g) were harvested in October 2014 from a local processing company in Shanghai (Song Jiang) and confined in a cultivation cabinet at a constant temperature (20℃) and humidity (90%) until natural death occurred. The death rate of the crabs was calculated daily. Different states of the crab, namely living and 0, 2, 5, 10, 15, and 24 hr postmortem were sampled for this study at 20℃. The crab was steamed at 100℃ for 20 min (Chen, Zhang, & Shrestha, 2007) then cooled to room temperature, after which the belly meat and roe were removed by hand, weighed, and the yield calculated. The samples were then stored at −80℃ until further analysis.

“Death” was determined once the animal's legs drooped naturally, and the eyes did not move when the animal was lifted by hand. The death rate (DR) was calculated as [DR (%) = (number of dead crabs/initial number of crabs) × 100].

The weight loss rate (WLR) was calculated as [WLR (%) = (weight loss/initial weight of crab) × 100].

The belly meat yield (MY) was calculated as [MY (%) = (weight of meat yield/weight of whole animal) × 100].

The crab roe yield (RY) was calculated as [RY (%) = (weight of crab roe/weight of whole animal) × 100].

### 2.2 Sensory evaluation

Ten trained panelists, five male and five female, ranging in age from 20 to 25 years, performed a sensory assessment of the uncooked crab quality according to ISO 8586 (2012). The sensory evaluation was conducted in reference to king crab (*Paralithodes camtschaticus*), with appropriate modifications for mitten crab (Lorentzen et al., 2014). Each tissue was evaluated based on the color of the unopened shell and the organizational structure of the opened shell. Scores were from 5 (best quality) to 1 (poorest quality) until the sample was rejected (Table 1). Each index weight was 0.3, 0.3, and 0.4, and the overall results were weighted for each index.

**TABLE 1:** Scale used for Chinese mitten crab sensory evaluation

	Description	Best (5 point)	Better (4 point)	Middle (3 point)	Worse (2 point)	Worst (1 point)
Crabs with unopened shells	Color and luster	Grayish with shine	Darker gray with shine	Grayish‐black with shine	Quite black with less shine	Crabs with unopened shells
	Odor	Natural smell very strong	Natural smell strong	Natural smell light, off‐odor slightly	No natural smell, smelly or ammonia smell	Strong stench or ammonia smell
Crabs with opened shells	Organizational Structure	Hepatopancreas and gonad tissue very tight	Hepatopancreas and gonad tissue tight	Hepatopancreas and gonad tissue less tight	Hepatopancreas and gonad tissue loose	Crabs with opened shells

### 2.3 Total volatile base nitrogen (TVBN)

TVBN was determined according to the method recommended by the European Commission Regulation (2005). Ten g portions of the uncooked samples were treated with 5 ml concentrated sulfuric acid and digested for 2 hr at 420℃, and then, samples were run through a Kjeldahl analyzer (FOSS Kjeltec8400). The concentration is expressed as mg N/100 g sample.

### 2.4 Biogenic amines

Biogenic amines were extracted and analyzed according to Özogul, Taylor, Quantick, and Özogul (2002) using an HPLC apparatus (W2690/5, Waters) and SUPELCOSIL LC‐18‐T, 250 × 4.6 mm, 5 µm column. All biogenic amine standards were purchased from Sigma‐Aldrich.

Crab meat (3.000 g) was homogenized in 10 ml of 0.4 M perchloric acid (PCA) for 2 min and centrifuged at 5,000 r/min for 15 min at 4℃. After centrifugation, the upper layer was decanted and transferred into a 25 ml brown flask and diluted. A 1 ml extraction was derivatizated by 100 µl of 2 mol/L NaOH, 300 µl of a saturated solution of NaHCO_3_, and 2 ml of 10 mg/ml dansyl chloride. All sample solutions were filtered through 0.22 µm filters prior to analysis, and then, 10 µl of the filtrate was injected into the HPLC. Two different eluents were used: (A) 0.01 mol/L ammonium acetate and (B) 90% acetonitrile in 0.01 mol/L ammonium acetate. The wavelength of the detector was set to UV 254 nm.

### 2.5 Microbiological analysis

Total viable counts (TVC) for the uncooked meats were analyzed as per the technique described by Zhou, Liu, Xie, and Wang (2011). Each belly meat or roe sample was picked by hand with gloves on a sanitized workbench. Uncooked belly meat and crab roe (10 g) were homogenized with 90 ml of normal saline (NS) containing 0.85% (w/v) NaCl, and then, the mixed solutions were serially diluted 10‐fold in the NS. One‐milliliter samples of each dilution were dispersed on a petri dish containing 15–20 ml agar medium, after which they were incubated at 30℃ for 72 ± 3 hr.

### 2.6 Volatile compound analysis

#### 2.6.1 Solid‐phase microextraction

A 65 µm carbowax/polydimethylsiloxane (CAR/PDMS) coated fused silica fiber was used as the solid phase. The coated fiber was inserted into a 20 ml headspace glass containing 2 g of homogenized sample and placed in a 90℃ water bath for 50 min. After adsorption, the fiber was immediately inserted into the GC–MS injector for 5 min and then removed.

#### 2.6.2 Gas chromatography conditions

An Agilent 6890 GC‐5975 mass selective detector (MSD) equipped with a DB‐5MS column (60 m length × 0.32 mm i.d. × 1 μm film thickness; Agilent Inc.) was used for the analysis. The initial oven temperature was 50℃ (no hold), followed by a 5℃/min linear ramp to 180℃ (no hold), and then an 8℃/min linear ramp to 240℃ (5 min hold). Helium (99.999%) was used as the carrier gas at a flow rate of 1 ml/min. The detector interface temperature was 250°C, the ion source temperature was 230°C, the ionization energy was 70 eV, the mass range was 40–450 a.m.u., the electron multiplier voltage was 1,576 V, and the scan rate was 1.8 s^−1^.

#### 2.6.3 Identification and quantitation

Volatiles were intendified by mass spectra matching (Wiley/NIST 2008 database) with compound matching greater than 800. The quantitation of compounds was according to the rentention index (RI).

RI was calculated as follows:

$$\text{RI}\;=\;\left(\frac{\text{Rt(x) - Rt(n)}}{\text{Rt(n + 1) - Rt(n)}}+n\right)\times 100.$$

where Rt(x) is the retention time of each volatile compound (x). Rt(n) and Rt(n + 1) are the retention times of the n‐alkanes eluting directly before and after the compound (x) under identical chromatographic conditions.

For quantification, 2,4,6‐trimethyl pyridine (TMP) was used as an internal standard, with 1 µg introduced into each 2 g homogenized sample.

The concentration of each volatile compound (Conc) was calculated as follows:

$$\text{Conc}\left(\text{ng}/g\right)=\frac{\text{Peak}\;\text{area}\;\text{ratio}\;(\text{compound}/\text{TMP})\times 1\;\mu g\;(\text{TMP})}{2g(\text{samples})}\times 1000$$

### 2.7 Statistical analysis

All measurements were conducted in triplicate. The data are presented as mean ± standard deviation (*SD*). SPSS 19.0 (SPSS Inc., Chicago, IL, USA) was used for the statistical analyses of the various indicators. Analysis of variance (ANOVA) was performed on the group means of the crab meat samples, and significant differences were tested at *p* ≤ .05. Changes in the meat content of each sample were mapped using SigmaPlot 12.5 (Systat Software Inc.) software.

## 3 RESULTS AND DISCUSSION

### 3.1 Death rate and yield

**FIGURE 1:**
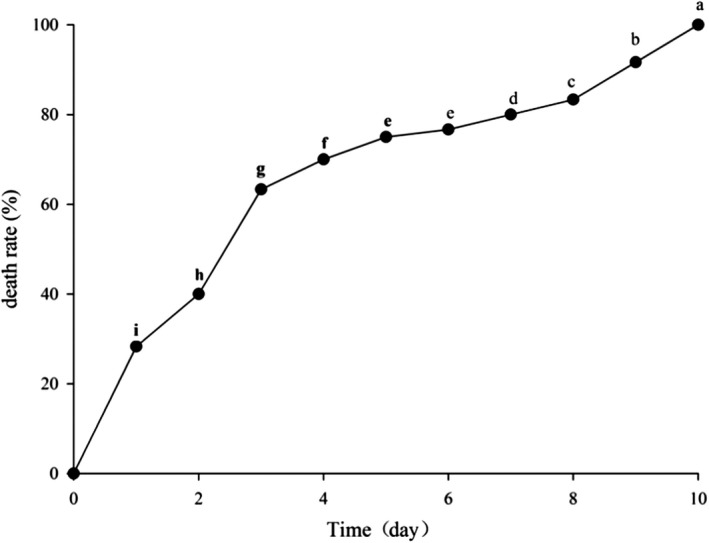
Cumulative death rate of *Eriocheir sinensis* during storage in a constant temperature and humidity incubator. Note: Different letters in the line indicate significant differences (*p* < .05)

The crabs died at varying rates as the storage time progressed, with the death rate being particularly high in the first three days. After storage in the cultivation cabinet at 20 ± 1℃ for 1 day, 28.33% of the crabs were dead; after 4 days, 70.00% of the crabs had died. The rate slowed thereafter, but all the crabs were dead by day 10 (Figure 1).

**FIGURE 2:**
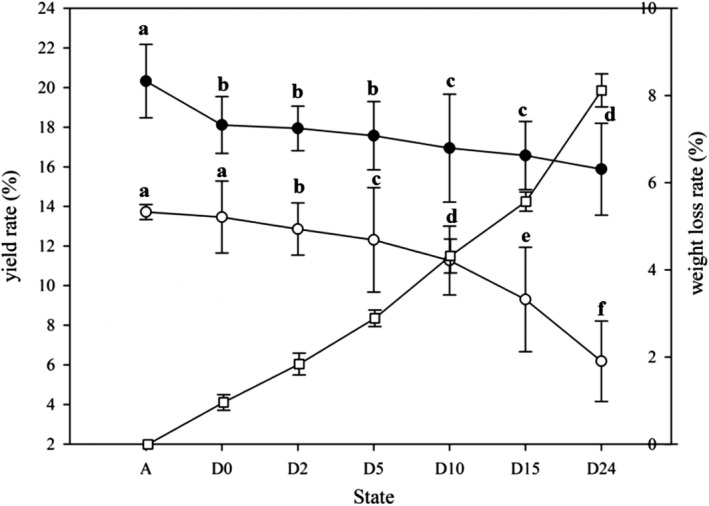
Belly meat yield (●), crab roe yield (o), and weight loss rate (□) of *E. sinensi*s during storage in different states, expressed as average content ± *SD*

The quality of the living crabs was considered the “initial quality,” with a weight loss rate of 0. The crab weight loss rate increased steeply to 8.18% by 24 hr postmortem (Figure 2), with the edible parts yield of *E. sinensi*s decreasing as the postmortem period increased. The yield for living crabs was generally similar to that reported by Wu et al. (2007) and Chen et al. (2007). The belly meat yield dropped sharply postmortem, and although the roe yield differed significantly between 0 and 2 hr postmortem, it had decreased dramatically to about 6% by 24 hr postmortem. The crab roe yield decreased at a faster rate than belly meat 5 hr postmortem, suggesting that the roe has a higher degradation rate overall.

### 3.2 Sensory evaluation

**FIGURE 3:**
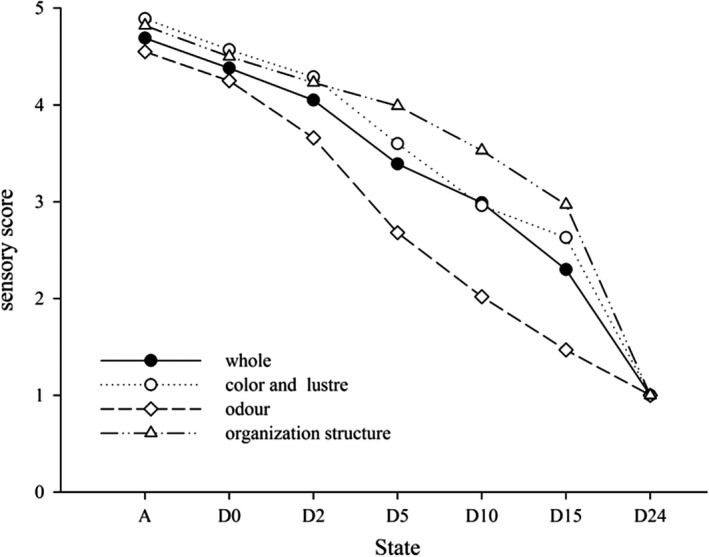
Sensory evaluation score of *E. sinensi*s during storage in different states using a 5‐point scale

Figure 3 shows the trends in the sensory evaluation scores of the Chinese mitten crabs as time progressed. The sensory scores declined as postmortem time increased. The shells of the living crabs were grayish and glossy, and they had well‐organized roe with a distinct, fresh odor, and no “off‐flavors.” As the length of time postmortem (or “death time”) increased, the crab shell gradually turned black and dull, while the roe structure began to loosen. By 24 hr postmortem, the crabs were rotting and producing a pungent, ammonia‐like odor. The layer of mucous membrane below the heart had become blurred and opaque. The crab samples’ sensory scores declined most rapidly in the first 5 hr postmortem. A strong odor was present by 10 hr postmortem, at which point the overall color score was 3.07, the odor score was 2.01, and the opened shells’ organizational structure score was 3.48. Rejection at this point was attributed to the pronounced ammoniac odor, although the crab paste inside the shell was still relatively well organized; the score for odor was lower than the overall score for shell color or paste inside the shell (Figure 3). These results demonstrate that the sensory score is primarily influenced by odor, with the other factors playing only a secondary role.

### 3.3 TVBN value

**FIGURE 4:**
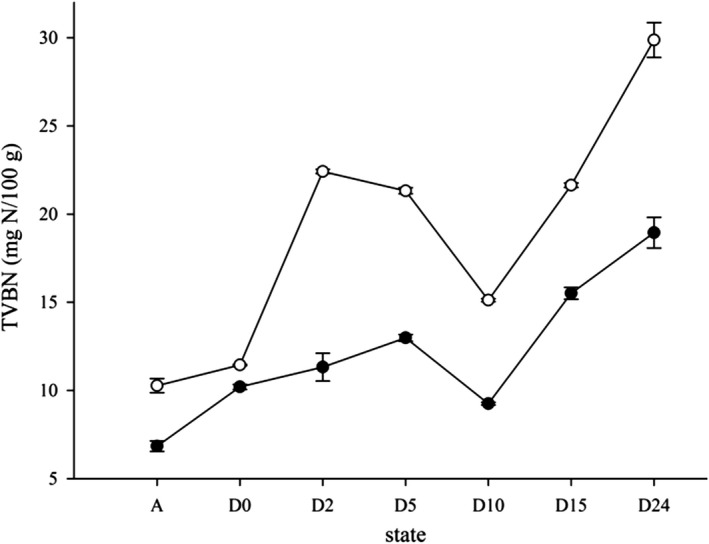
Changes in belly meat TVBN (●) and crab roe TVBN(o) of *E. sinensi*s during storage in different states expressed as an average score ± *SD*

The TVBN value, a common indicator of spoilage, increased and then decreased as death time increased, which differs from the trends reported for other seafood products such as fish or shrimp. Previous studies on wild white grouper (Özogul, Özogul, & Kuley, 2008), Pacific white shrimp (Okpala, Choo, & Dykes, 2014), and snow crab (Lorentzen, Rotabakk, Olsen, Skuland, & Siikavuopio, 2016) all showed TVBN values that gradually increased with increasing storage time. However, Anacleto et al. (2011) reported highly variable changes in TVBN in 24 hr postmortem cooked crab refrigerated at 4℃, and our results (Figure 4) confirm this trend. The sharp decline in TVBN at 10 hr may have been due to the loss of water and the generation of certain volatile amines. TVBN was higher in the roe than the belly meat, suggesting a higher level of degradation in the former. This may be attributable to the high content of free amino acids, protein, and nonprotein nitrogen in the roe (Lorentzen et al., 2016; Wu et al., 2010).

The European Commission has set the legal limit for TVBN in seafood in the range from 25 to 35 mgN/100 g (EC, 2005). The TVBN levels of belly meat and crab roe in our samples at 24 hr postmortem were 29.89 mgN/100 g 18.95 TVBN mgN/100 g, respectively, which do not exceed the legal limit. However, TVBN is not an accurate spoilage indicator for cooked Chinese mitten crab quality evaluation, nor for other crustaceans (Huidobro et al., 2002).

### 3.4 Biogenic amine

Table 2 shows the biogenic amine content of the Chinese mitten crab samples at different times postmortem. The biogenic amines found in crab mainly consist of putrescine, cadaverine, tyramine, and small amounts of spermidine and spermine. In previous studies, histamine—an important indicator of aquatic toxicity and hygienic quality—has been found to be present even immediately after catching the animal (Lehane & Olley, 2000; Mah, Han, Oh, Kim, & Hwang, 2002). However, no histamine was detected in any of the crabs, from living to 24 hr postmortem, tested in this study. (Özogul et al., 2008) similarly did not detect histamine in white grouper during storage on ice; other studies have reported similar phenomena in deep‐water pink shrimp and Eastern Atlantic grouper (Huidobro et al., 2002; Maggio, Andaloro, Hemida, & Arculeo, 2005). Shakila, Vijayalakshmi, and Jeyasekaran (2003) have argued that putrescine and cadaverine are toxicity potentiators for histamine, and there are different typical biogenic amines in different aquatic products.

**TABLE 2:** Changes in the amount of biogenic amine extracted from crabs in different states

State	Putrescine		Cadaverine		Tyramine		Spermidine		Spermine	
	BM	CR	BM	CR	BM	CR	BM	CR	BM	CR
D5	6.82 ± 2.04^a^	N.D.	N.D.	N.D.	3.76 ± 0.85^a^	N.D.	N.D.	N.D.	N.D.	N.D.
D10	15.06 ± 2.04^b^	9.14 ± 2.60^a^	4.33 ± 1.15^a^	7.40 ± 0.52^a^	4.02 ± 0.75^a^	6.59 ± 0.98^a^	3.09 ± 0.60^a^	N.D.	N.D.	3.59 ± 1.03a
D15	102.45 ± 9.04^c^	146.78 ± 13.62^b^	15.10 ± 2.81^b^	10.03 ± 3.38^b^	9.45 ± 2.10^b^	8.37 ± 1.22^a^	N.D	N.D.	3.59 ± 1.03a	3.05 ± 1.21a
D24	197.72 ± 11.21^d^	218.66 ± 9.83^c^	25.71 ± 4.17^c^	27.84 ± 2.36^c^	21.42 ± 6.51^c^	27.59 ± 5.81^b^	2.97 ± 0.77^a^	3.15 ± 0.27^a^	4.01 ± 0.76a	5.25 ± 0.76b

Note

Values are presented as means ± *SD*. Unit: mg/kg. Three replications tested. D5, D10, D15, and D24 indicate 5, 10, 15, and 24 hr postmortem, respectively.

Abbreviations: BR, belly meat; CR, crab roe.

Different superscript letters in the same column indicate a significant difference *p* < .05.

No biogenic amines were detected in the living, 0 hr, or 2 hr dead crab samples. The amount of putrescine, cadaverine, and tyramine increased from 5 hr postmortem onwards, with the putrescine content increasing faster than either cadaverine or tyramine. This may be related to the content of amino acids and the precursors of putrescine, cadaverine, and tyramine: ornithine, lysine, and tyrosine, respectively (Anacleto et al., 2011).

The putrescine, cadaverine, and tyramine content in the belly meat reached 197.72 mg/100 g, 25.71 mg/100 g, and 21.42 mg/100 g and in the roe reached 218.66 mg/100 g, 27.84 mg/100 g, and 27.59 mg/100 g, respectively, at 24 hr postmortem (Table 2). None of the levels of biogenic amines were sufficient to cause poisoning; the histamine limit in food is 50 mg/kg (FDA), and the LD50 (median lethal dose) for putrescine and cadaverine are 1,750 mg/kg and 270 mg/kg, respectively (Oguri et al., 2007; Sandler, 1974; Shalaby, 1996), but the crab's unacceptable sensory score was primarily caused by these biogenic amines.

### 3.5 Microbial analysis

**FIGURE 5:**
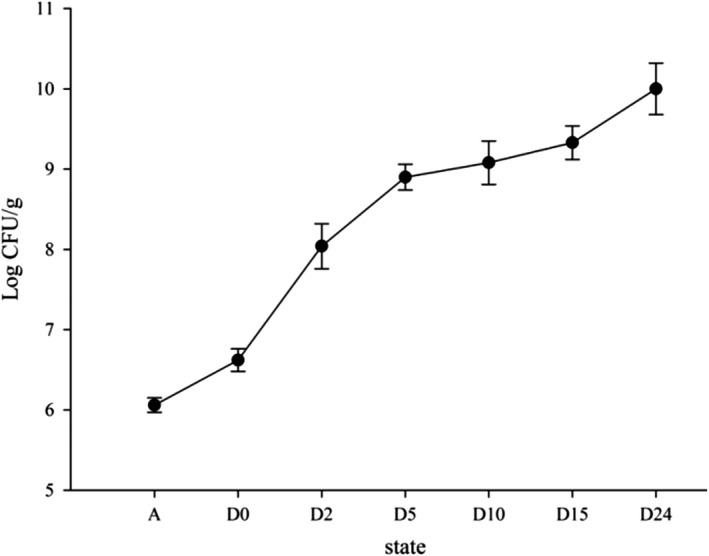
Microbiological growth of raw Chinese Mitten Crab in different states

The initial TVC (6.06 Log CFU/g) of the living crab samples (Figure 5) represented a very high background content compared with those reported for raw snow crab (2.5 Log CFU/g), raw blue crab meat (2.5 Log CFU/g), or fresh puffer fish (2.80 Log CFU/g) (Lorentzen et al., 2016; Sarnoski et al., 2010; Zhou et al., 2011). The high nutrient content and loose connection between collagen fibers provide a rich environment for microbial growth (Suyama & Konosu, 1987).

TVC increased quickly immediately postmortem (6.62 Log CFU/g) to 5 hr postmortem (8.90 Log CFU/g) and then continued to increase slowly until it reached 10.00 Log CFU/g at 24 hr postmortem. The sudden increase in microbial growth at death corresponds closely to the belly meat yield and sensory evaluation results. (Özogul, Polat, & Özogul, 2004) observed similar trends in gutted sardines. Upon death, the crab's immune system collapses resulting in rapid microbial growth. In raw meat, Huang, Shiau, Hung, and Hwang (2006) reported that 5.00 Log CFU/g is the maximum permissible level for human consumption. Microbiological acceptability is also an indicator of shelf life in iced gutted turbot (Özogul, Gökbulut, Özogul, & Özyurt, 2006). In our study, the TVC of the living crab was already above the permissible limit.

### 3.6 Volatile compounds

Sixty‐six volatile compounds were identified in the belly meat and roe in different states (Table 3). These were 23 hydrocarbons, 11 alcohols, 4 ketones, 17 aldehydes, 5 N‐containing compounds, 4 S‐containing compounds, and 2 “others.” Among the 66 volatile compounds, 47 were found in the belly meat and 48 in the roe.

**TABLE 3:** Changes in the content of volatile compounds extracted from crabs in different states

Number	Compounds	Means of identification	Belly meat							Crab roe						
			A	D0	D2	D5	D10	D15	D24	A	D0	D2	D5	D10	D15	D24
	Hydrocarbons															
W1	Undecane	MS,RI	144.92 ± 16.9	75.12 ± 9.06	43.37 ± 10.83	N.D.	N.D.	N.D.	N.D.	N.D.	N.D.	N.D.	N.D.	N.D.	N.D.	N.D.
W2	Decane, 1,1'‐oxybis‐	MS,RI	N.D.	N.D.	N.D.	N.D.	N.D.	N.D.	N.D.	76.82 ± 15.84	52.89 ± 46.2	N.D.	N.D.	N.D.	N.D.	N.D.
W3	Dodecane	MS,RI	708.06 ± 91.96	624.29 ± 23.53	427.81 ± 17.73	210.63 ± 26.29	116.84 ± 10.03	85.28 ± 29.13	68.78 ± 10.60	1,270.00 ± 205.69	892.45 ± 28.55	824.25 ± 150.88	727.79 ± 157.29	633.60 ± 120.52	428.42 ± 15.0.39	345.78 ± 32.49
W4	1‐Tridecene	MS,RI	135.66 ± 18.16	104.38 ± 19.17	60.29 ± 9.86	N.D.	N.D.	N.D.	N.D.	N.D.	N.D.	N.D.	N.D.	N.D.	N.D.	N.D.
W5	Tridecane	MS,RI	1,242.10 ± 111.44	1,067.83 ± 99.51	852.38 ± 101.00	537.29 ± 86.52	231.77 ± 11.67	184.64 ± 21.43	121.77 ± 14.28	N.D.	N.D.	N.D.	N.D.	N.D.	N.D.	N.D.
W6	Heptylcyclohexane	MS	101.46 ± 12.51	87.39 ± 13.02	64.72 ± 14.34	43.37 ± 17.39	N.D.	68.38 ± 10.31	83.26 ± 9.42	89.04 ± 13.35	132.23 ± 50.3	N.D.	N.D.	44.32 ± 3.52	63.82 ± 6.37	99.95 ± 4.51
W7	Tetradecane, 4‐methyl	MS,RI	141.93 ± 16.02	135.27 ± 9.83	86.49 ± 20.17	63.28 ± 8.47	24.49 ± 6.31	85.31 ± 6.35	165.38 ± 15.39	116.44 ± 25.25	N.D.	N.D.	N.D.	61.63 ± 5.17	102.38 ± 8.05	164.24 ± 9.94
W8	Tridecane, 3‐methylene‐	MS,RI	44.73 ± 5.53	20.36 ± 7.73	N.D.	N.D.	N.D.	N.D.	N.D.	N.D.	N.D.	N.D.	N.D.	N.D.	N.D.	N.D.
W9	3‐Tetradecene, (E)‐	MS,RI	112.31 ± 13.66	79.37 ± 12.58	27.69 ± 10.03	N.D.	N.D.	N.D.	N.D.	N.D.	N.D.	N.D.	N.D.	N.D.	N.D.	N.D.
W10	Tetradecane	MS	339.16 ± 10.37	233.27 ± 27.51	N.D.	103.38 ± 9.23	71.95 ± 10.56	53.31 ± 17.39	N.D.	N.D.	N.D.	N.D.	N.D.	N.D.	N.D.	N.D.
W11	Tetradecane, 2,6,10‐trimethyl	MS,RI	68.73 ± 1.09	127.31 ± 5.26	28.65 ± 2.94	N.D.	N.D.	N.D.	N.D.	73.55 ± 17.74	121.76 ± 47.41	97.88 ± 4.72	N.D.	N.D.	103.28 ± 13.92	87.57 ± 1.86
W12	Hexadecane, 2,6,10,14‐tetramethyl‐	MS,RI	227.37 ± 32.67	349.29 ± 17.42	265.72 ± 25.16	187.21 ± 14.37	121.5 ± 19.53	74.51 ± 20.15	36.29 ± 8.47	N.D.	N.D.	N.D.	N.D.	N.D.	N.D.	N.D.
W13	1‐Pentadecene	MS,RI	206.74 ± 10.96	164.52 ± 20.27	121.92 ± 13.41	62.38 ± 15.62	N.D.	N.D.	N.D.	N.D.	N.D.	N.D.	N.D.	N.D.	N.D.	N.D.
W14	Pentadecane	MS,RI	178.15 ± 10.35	132.47 ± 12.38	154.27 ± 7.25	100.43 ± 17.21	85.41 ± 34.75	N.D.	47.52 ± 18.06	478.44 ± 44.52	265.63 ± 129.18	397.71 ± 38.69	361.76 ± 25.41	98.35 ± 12.24	136.59 ± 9.58	372.08 ± 42.79
W15	*n*‐Nonylcyclohexane	MS,RI	234.12 ± 7.52	234.12 ± 7.53	234.12 ± 7.54	234.12 ± 7.55	N.D.	N.D.	N.D.	N.D.	N.D.	N.D.	N.D.	N.D.	N.D.	N.D.
W16	Pentadecane, 3‐methyl‐	MS,RI	129.98 ± 8.87	64.21 ± 5.42	42.59 ± 8.25	N.D.	N.D.	N.D.	N.D.	N.D.	N.D.	N.D.	N.D.	N.D.	N.D.	N.D.
W17	Hexadecane	MS,RI	268.90 ± 18.24	136.42 ± 12.31	98.99 ± 2.86	69.20 ± 14.29	59.58 ± 13.39	36.57 ± 8.51	N.D.	N.D.	N.D.	N.D.	N.D.	N.D.	N.D.	N.D.
W18	Cyclopentane, undecyl‐	MS,RI	108.79 ± 5.97	78.63 ± 8.67	97.35 ± 11.70	67.42 ± 7.12	35.27 ± 2.07	N.D.	N.D.	N.D.	N.D.	N.D.	N.D.	N.D.	N.D.	N.D.
W19	1‐Eicosene	MS,RI	66.67 ± 10.42	43.66 ± 8.26	N.D.	N.D.	N.D.	N.D.	N.D.	N.D.	N.D.	N.D.	N.D.	N.D.	N.D.	N.D.
W20	Pentadecane, 2,6,10,14‐tetramethyl‐	MS	1,377.32 ± 118.09	1,028.52 ± 72.91	1,472.01 ± 114.27	1,007.32 ± 92.32	948.6 ± 170.89	872.81 ± 189.02	664.02 ± 52.14	2,914.88 ± 1,337.25	2,338.44 ± 1,182.11	2,103.31 ± 81.6	2003.31 ± 81.6	1875.23 ± 152.04	1745.17 ± 102.47	1675.91 ± 92.4
W21	Heptacosane	MS,RI	49.04 ± 13.23	143.81 ± 16.84	148.92 ± 18.43	103.93 ± 9.41	95.46 ± 9.44	63.65 ± 8.92	54.21 ± 10.37	N.D.	N.D.	N.D.	N.D.	N.D.	N.D.	N.D.
W22	Cyclohexane, undecyl‐	MS,RI	59.00 ± 5.41	20.62 ± 4.37	N.D.	N.D.	N.D.	N.D.	N.D.	N.D.	N.D.	N.D.	N.D.	N.D.	N.D.	N.D.
W23	Oxirane, hexadecyl‐	MS,RI	N.D.	N.D.	N.D.	N.D.	N.D.	N.D.	N.D.	534.32 ± 39.63	N.D.	906.98 ± 110.81	906.98 ± 110.81	337.08 ± 62.9	N.D.	N.D.
Subtotal			5,945.14 ± 539.37	4,950.86 ± 413.58	4,227.29 ± 395.77	2,789.96 ± 325.79	1,790.87 ± 288.64	1524.46 ± 311.21	1,452.59 ± 113.92	5,033.67 ± 393.114	4,805.65 ± 338.34	4,007.74 ± 1644.46	3,980.95 ± 1,660.89	3,362.39 ± 268.36	3,079.66 ± 140.39	2,992.20 ± 180.05
	Alcohol															
C1	1‐Octen‐3‐ol	MS,RI	N.D.	N.D.	N.D.	136.48 ± 12.52	62.04 ± 16.30	N.D.	N.D.	216.55 ± 51.97	161.55 ± 96.92	N.D.	59.77 ± 34.51	N.D.	N.D.	N.D.
C2	2‐Octen‐1‐ol	MS	N.D.	N.D.	N.D.	N.D.	N.D.	N.D.	N.D.	42.56 ± 17.47	N.D.	N.D.	N.D.	N.D.	N.D.	N.D.
C3	2‐Octyn‐1‐ol	MS,RI	N.D.	N.D.	N.D.	N.D.	N.D.	N.D.	N.D.	233.16 ± 144.12	36.58 ± 63.36	34.9 ± 15.86	N.D.	N.D.	N.D.	N.D.
C4	4‐nonanol	MS,RI	N.D.	N.D.	N.D.	N.D.	208.8 ± 8.53	153.62 ± 10.26	83.61 ± 15.17	N.D.	N.D.	129.06 ± 18.17	N.D.	58.22 ± 5.11	N.D.	N.D.
C5	3‐Decen‐1‐ol, (Z)‐	MS,RI	N.D.	321.83 ± 23.47	251.82 ± 18.26	217.28 ± 24.51	183.11 ± 10.86	93.71 ± 16.27	61.35 ± 9.21	N.D.	N.D.	N.D.	N.D.	N.D.	N.D.	N.D.
C6	10‐undecenol	MS,RI	N.D.	N.D.	N.D.	N.D.	342.19 ± 39.21	210.52 ± 18.31	152.24 ± 17.36	N.D.	980.17 ± 85.75	1,351.56 ± 37.53	N.D.	748.05 ± 52.19	521.20 ± 15.10	221.72 ± 56.75
C7	6‐Pentadecen‐1‐ol, (Z)‐	MS,RI	N.D.	N.D.	N.D.	N.D.	N.D.	N.D.	N.D.	N.D.	N.D.	N.D.	137.33 ± 22.02	N.D.	N.D.	N.D.
C8	1‐Hexadecanol, 2‐methyl‐	MS,RI	129.40 ± 12.27	218.41 ± 17.28	153.92 ± 13.71	32.51 ± 17.28	N.D.	N.D.	N.D.	N.D.	N.D.	N.D.	N.D.	N.D.	N.D.	N.D.
C9	1‐Dodecanol, 3,7,11‐trimethyl‐	MS	50.48 ± 4.66	27.27 ± 2.34	N.D.	N.D.	N.D.	N.D.	N.D.	N.D.	N.D.	N.D.	N.D.	24.82 ± 7.47	56.28 ± 5.63	68.48 ± 7.47
C10	3,7,11,15‐Tetramethyl‐2‐hexadecen‐1‐ol	MS,RI	70.23 ± 8.4	73.01 ± 6.43	42.90 ± 7.31	N.D.	N.D.	N.D.	N.D.	N.D.	N.D.	N.D.	N.D.	20.06 ± 14.11	35.17 ± 5.72	N.D.
C11	1‐Heptacosanol	MS,RI	N.D.	N.D.	N.D.	N.D.	N.D.	N.D.	N.D.	N.D.	N.D.	N.D.	N.D	33.49 ± 2.46	26.92 ± 3.54	N.D.
Subtotal			250.11 ± 25.33	640.52 ± 49.52	448.64 ± 39.28	386.27 ± 54.31	796.14 ± 74.9	457.85 ± 44.84	297.2 ± 41.74	492.27 ± 123.56	1,351.56 ± 37.53	1,351.56 ± 37.53	197.10 ± 56.53	985.74 ± 95.57	639.57 ± 29.99	290.2 ± 64.22
	Ketone															
T1	2‐Butanone	MS,RI	N.D.	N.D.	N.D.	N.D.	N.D.	N.D.	N.D.	27.14 ± 5.79	19.82 ± 6.48	N.D	N.D	N.D	N.D	N.D
T2	2‐Nonanone	MS,RI	N.D.	N.D.	N.D.	N.D.	48.96 ± 10.64	24.91 ± 8.02	N.D.	N.D.	92.14 ± 12.87	N.D.	N.D.	53.52 ± 17.95	42.03 ± 7.42	N.D.
T3	5,9‐Undecadien‐2‐one, 6,10‐dimethyl‐, (E)‐		57.2 ± 11.24	86.27 ± 12.62	153.01 ± 16.39	62.03 ± 9.27	57.2 ± 11.24	86.27 ± 12.62	153.01 ± 16.39	N.D.	N.D.	N.D.	N.D.	N.D.	N.D.	N.D.
T4	3‐Buten‐2‐one, 4‐(2,6,6‐trimethyl‐1‐cyclohexen‐1‐yl)‐	MS	N.D.	N.D.	N.D.	N.D.	N.D.	N.D.	N.D.	78.08 ± 8.75	87.95 ± 25.44	N.D.	N.D.	N.D.	N.D.	N.D.
Subtotal			57.2 ± 11.24	86.27 ± 12.62	153.01 ± 16.39	62.03 ± 9.27	90.65 ± 20.16	112.84 ± 19.63	153.01 ± 16.39	106.24 ± 14.91	200.01 ± 40.31	N.D.	N.D.	53.52 ± 17.95	26.41 ± 6.52	N.D.
	Aldehyde									.						
Q1	Benzaldehyde	MS,RI	N.D.	N.D.	N.D.	N.D.	N.D.	N.D.	N.D.	N.D.	237.06 ± 136.86	216.02 ± 9.83	231.87 ± 26.14	3.06 ± 1.76	15.79 ± 3.75	23.23 ± 3.04
Q2	Benzeneacetaldehyde	MS,RI	N.D.	N.D.	N.D.	N.D.	N.D.	N.D.	N.D.	80.60 ± 19.24	161.14 ± 62.07	164.58 ± 48.54	175.53 ± 23.28	56.70 ± 3.82	56.70 ± 3.83	104.83 ± 3.25
Q3	2‐Octenal	MS,RI	N.D.	N.D.	N.D.	N.D.	N.D.	N.D.	N.D.	77.10 ± 21.66	80.75 ± 37.43	N.D.	N.D.	26.23 ± 2.34	N.D.	N.D.
Q4	Nonanal	MS,RI	481.24 ± 50.53	637.38 ± 60.43	748.73 ± 58.79	497.38 ± 38.52	428.19 ± 34.79	206.47 ± 42.58	174.38 ± 32.38	372.37 ± 50.08	640.63 ± 163.22	N.D.	N.D.	363.59 ± 29.67	257.15 ± 20.86	N.D.
Q5	2,6‐Nonadienal	MS	N.D.	N.D.	N.D.	N.D.	N.D.	N.D.	N.D.	119.74 ± 25.91	137.04 ± 27.32	134.47 ± 9.5	121.77 ± 21.9	73.65 ± 1.36	52.17 ± 6.28	N.D.
Q6	cis‐4‐Decenal	MS,RI	N.D.	N.D.	N.D.	N.D.	52.15 ± 2.24		N.D.	50.97 ± 9.29	110.90 ± 38.3	115.86 ± 10.78	N.D.	51.63 ± 2.71	N.D.	N.D.
Q7	Decanal	MS,RI	132.95 ± 2.84	157.58 ± 31.09	204.47 ± 22.21	187.31 ± 25.71	158.47 ± 22.21	85.53 ± 21.71	N.D.	115.29 ± 18.81	149.19 ± 47.54	215.82 ± 52.75	211.37 ± 75.44	93.42 ± 5.23	37.35 ± 4.17	N.D.
Q8	2‐Decenal	MS,RI	N.D.	N.D.	N.D.	N.D.	N.D.	N.D.	N.D.	66.52 ± 31.04	N.D.	N.D.	N.D.	51.60 ± 8.11	N.D.	N.D.
Q9	Undecanal	MS,RI	69.24 ± 4.13	87.29 ± 12.71	105.63 ± 18.42	145.48 ± 10.35	117.68 ± 12.35	75.42 ± 14.36	65.17 ± 8.54	78.75 ± 16.14	86.56 ± 18.36	111.80 ± 6.98	96.87 ± 9.13	84.59 ± 8.05	27.27 ± 4.26	N.D.
Q10	2,4‐Decadienal	MS	N.D.	N.D.	N.D.	N.D.	N.D.	N.D.	N.D.	75.48 ± 18.87	N.D.	N.D.	N.D.	N.D.	N.D.	N.D.
Q11	2‐Undecenal	MS,RI	N.D.	N.D.	N.D.	N.D.	N.D.	N.D.	N.D.	138.14 ± 34.22	N.D.	N.D.	N.D.	N.D.	N.D.	N.D.
Q12	7‐Hexadecenal	MS,RI	N.D.	N.D.	N.D.	N.D.	N.D.	N.D.	N.D.	29.03 ± 8.19	N.D.	N.D.	N.D.	N.D.	N.D.	N.D.
Q13	Dodecanal	MS,RI	41.04 ± 4.98	63.28 ± 8.02	71.93 ± 12.03	52.47 ± 6.43	34.62 ± 6.16	40.53 ± 12.42	64.22 ± 9.84	83.17 ± 10.87	74.85 ± 31.96	90.02 ± 9.5	79.93 ± 2.84	55.21 ± 5.82	54.68 ± 6.21	84.73 ± 53.63
Q14	Tridecanal	MS,RI	N.D.	N.D.	N.D.	N.D.	N.D.	N.D.	N.D.	93.09 ± 12.68	74.06 ± 51.06	N.D.	67.12 ± 7.63	33.58 ± 9.87	2036 ± 10.27	N.D.
Q15	Tetradecanal	MS,RI	45.70 ± 3.36	78.29 ± 8.62	104.83 ± 17.42	65.39 ± 6.27	36.06 ± 7.78	26.41 ± 3.52	N.D.	66.61 ± 4.64	156.86 ± 70.46	185.79 ± 3.48	126.98 ± 19.99	75.16 ± 11.84	60.26 ± 7.25	55.77 ± 11.12
Q16	Hexadecanal	MS,RI	183.09 ± 4.32	317.26 ± 15.31	559.47 ± 23.18	486.19 ± 16.28	212.02 ± 66.93	196.53 ± 13.27	137.39 ± 27.64	624.51 ± 60.98	1,243.98 ± 81.04	3,795.4 ± 175.44	2,819.65 ± 294.04	1,434.83 ± 161.42	1,328.59 ± 92.25	1,278.34 ± 130.04
Q17	Octadecanal	MS,RI	123.31 ± 12.32	167.93 ± 25.37	186.28 ± 18.02	97.29 ± 12.54	51.60 ± 13.01	63.38 ± 17.31	48.18 ± 9.27	306.89 ± 40.84	282.79 ± 239.18	1,078.57 ± 392.65	146.16 ± 128.38	219.73 ± 64.54	278.24 ± 14.52	321.94 ± 30.45
Subtotal			1,076.57 ± 82.48	1509.01 ± 161.55	2,134.35 ± 186.46	1593.54 ± 125.37	1,180.90 ± 179.01	758.91 ± 139.5	489.34 ± 87.67	2,378.26 ± 142.18	3,435.18 ± 256.24	5,642.80 ± 239.18	4,028.82 ± 392.65	2,622.98 ± 128.38	4,912.21 ± 217.58	1875.16 ± 123.61
N‐containing compounds																
N1	Trimethylamine	MS,RI	427.42 ± 19.27	1,740.21 ± 42.52	4,145.94 ± 146.85	5,974.59 ± 217.45	6,386.93 ± 218.36	7,328.58 ± 416.48	8,328.48 ± 743.27	N.D.	N.D.	142.61 ± 12.17	489.60 ± 40.27	942.74 ± 47.16	1549.64 ± 58.39	2,242.61 ± 52.17
N2	Oxime‐, methoxy‐phenyl‐	MS	366.66 ± 79.07	325.64 ± 39.38	312.39 ± 28.46	296.39 ± 24.82	291.53 ± 160.17	182.38 ± 37.31	112.58 ± 38.02	1,289.71 ± 44.78	1,135.71 ± 87.01	1,074.63 ± 53.15	879.71 ± 29.67	692.59 ± 44.78	702.46 ± 62.03	673.56 ± 66.60
N3	1‐Butanamine, N‐butyl‐		496.24 ± 316.5	672.24 ± 69.36	536.82 ± 42.03	496.24 ± 316.8	595.91 ± 24.27	621.49 ± 52.06	548.28 ± 90.45	N.D.	N.D.	N.D.	N.D.	N.D.	N.D.	N.D.
N4	Formamide, N,N‐dibutyl‐	MS	N.D.	N.D.	N.D.	N.D.	N.D.	N.D.	N.D.	35.29 ± 1.78	113.42 ± 14.13	180.61 ± 6.6.8	N.D.	53.80 ± 2.14	75.54 ± 9.48	84.99 ± 7.46
N5	Indole	MS	N.D.	18.28 ± 3.93	22.64 ± 4.86	38.35 ± 8.75	86.79 ± 7.42	149.97 ± 10.36	328.47 ± 16.49	N.D.	N.D.	N.D.	N.D.	23.61 ± 8.37	35.74 ± 5.54	49.17 ± 8.39
S‐containing compounds																
S1	Dimethyl sulfide	MS,RI	N.D.	35.73 ± 7.45	74.92 ± 9.43	123.04 ± 11.38	285.83 ± 14.70	402.84 ± 32.67	864.03 ± 120.36	N.D.	N.D.	N.D.	279.49 ± 17.39	292.57 ± 15.30	469.38 ± 20.31	800.37 ± 21.05
S2	Dimethyldisulfide	MS	N.D.	N.D.	N.D.	37.07 ± 3.85	86.43 ± 10.49	178.38 ± 26.07	386.64 ± 41.93	N.D.	N.D.	N.D.	N.D.	72.52 ± 9.06	103.37 ± 12.48	255.39 ± 32.72
S3	Dimethyltrisulfide	MS	N.D.	N.D.	N.D.	N.D.	42.86 ± 9.65	74.85 ± 6.47	121.58 ± 12.65	N.D.	N.D.	N.D.	N.D.	37.58 ± 8.32	58.15 ± 10.32	85.46 ± 19.44
S4	Tetrasulfide, dimethyl	MS,RI	N.D.	N.D.	N.D.	N.D.	28.73 ± 5.72	35.96 ± 6.74	47.93 ± 6.68	N.D.	N.D.	N.D.	N.D.	14.80 ± 3.38	19.27 ± 4.92	24.74 ± 4.63
	Others															
Z1	3‐(1‐Cyclopentenyl) furan	MS,RI	N.D.	N.D.	N.D.	N.D.	N.D.	N.D.	N.D.	54.83 ± 31.7	N.D.	N.D.	N.D.	N.D.	N.D.	N.D.
Z2	Butylated Hydroxytoluene	MS,RI	144.09 ± 22.2	253.27 ± 17.39	336.71 ± 28.79	187.96.09 ± 14.83	90.39 ± 14.38	83.53 ± 16.93	63.07 ± 14.39	35.29 ± 1.78	113.42 ± 14.13	180.61 ± 6.6.8	N.D.	53.80 ± 2.14	63.28 ± 12.61	84.99 ± 7.46

Note

Values are presented as means ± *SD*. Unit: µg/kg. Three replications tested. A, D0, D2, D5, D10, D15, and D24 indicate alive, 0, 2, 5, 10, 15, and 24 hr postmortem, respectively.

In column 1: W stands for Alkanes, C stands for Alcohols, T stands for: Acetones, Q stands for: Aldehydes, N stands for: Nitrogen‐containing compounds, S stands for: Sulfur‐containing compounds and Z stands for: other compounds.

Hydrocarbons usually make only a minimal contribution to odor due to their high odor thresholds, and they only affect the flavor at relatively high contents (Gu et al., 2013). Hydrocarbon contents decreased as death time increased, dropping from 5,945.14 ng/g to 1,452.59 ng/g in the belly meat and from 5,033.67 ± 393.114 ng/g to 2,992.20 ± 180.05 ng/g in the roe, indicating that the aroma gradually decreased as the product aged. Dodecane and 2,6,10,14‐tetramethyl‐pentadecane made up nearly half of the 23 hydrocarbons, similar to results reported by Gu et al. (2013) and Chen and Zhang (2010). The dodecane decreased from 25.33% to 10.50%, while the 2,6,10,14‐tetramethyl‐pentadecane saw less of a decline, dropping from 57.90% to 50.78% as a percentage of the total hydrocarbon content.

Due to their high odor thresholds, alcohols also usually contribute little to the overall flavor unless they are present at very high levels or contain unsaturated bonds. The alcohol volatility changed as time after death increased. Small molecular weight alcohols (1‐Octen‐3‐ol; 2‐Octen‐1‐ol; 2‐Octyn‐1‐ol) were identified in the roe within 5 hr postmortem, and large molecular weight alcohols (3,7,11‐trimethyl‐; 3,7,11,15‐Tetramethyl‐2‐hexadecen‐1‐ol; 1‐Heptacosanol) were identified in 10 hr postmortem. This trend was not observed in the belly meat, however. This may be associated with the production of alcohols caused by sugar degradation and fat oxidation.

A low ketone content was also identified: 2‐Nonanone was observed at 10 and 15 hr postmortem for belly meat, and 3‐Buten‐2‐one, 4‐(2,6,6‐trimethyl‐1‐cyclohexen‐1‐yl) was observed in living crab and 0 hr postmortem in the roe.

Aldehydes have low odor thresholds and typically form due to fat degradation, making them effective indicators of aroma in seafood products. Among the 17 aldehydes present in the samples, 7 were found in the belly meat and 17 in the roe. The content increased and then decreased with increasing time postmortem. For belly meat, cis‐4‐Decenal was only identified 10 hr postmortem and 2,4‐Decadienal, 2‐Undecenal, and 7‐Hexadecenal only in the living crab roe. These results may be associated with aldehyde production; amino acid Strecker reactions and lipid oxidative degradation are the two main sources of aldehydes in crustaceans (Baek & Cadwallader, 1997).

N‐ and S‐containing compounds are also known to contribute to aquatic aromas. We found five N‐containing compounds in our samples, including TMA and indole. Pure TMA is associated with ammonia‐like odors that are not present in very fresh seafood. It is typically found together with acid and pyridine, which are responsible for “fishy” odors. Oxime‐ and methoxy‐phenyl‐, which are associated with mildewy and meaty odors, gradually decreased as death time increased; 1‐Butanamine and N‐butyl‐ were only found in the belly meat, while formamide and N,N‐dibutyl‐ were only detected in the roe.

TMA commonly serves as an indicator of spoilage in aquatic products. The content of TMA sharply increased as time after death increased. Other researchers have also reported this trend: TMA levels rose from 0.43 µg/g (in the living samples) to 8.33 µg/g (at 24 hr postmortem) for cooked blue crab stored at 4℃, 40.80 µg/g (at 8 days) and 30.00 µg/g (7 days) for wild white grouper stored on ice (Özogul et al., 2008; Sarnoski et al., 2010). There was a significant increase from living to 2 hr postmortem, similar to reports by Sarnoski et al. (2010) for blue crab stored at 4℃, who considered TMA as a sensitive indicator of early spoilage in crab meat. In the crab roe, TMA was identified at 2 hr postmortem but at significantly lower levels than in the belly meat. At 10 hr postmortem, the crab produced a significant odor that was likely due to the increasing TMA content.

Indole has been used as an indicator of sepsis in shrimp (Snellings et al., 2003). In this study, indole was identified at 0 hr (belly meat) and 10 hr (roe) postmortem. There was no significant difference in the amount of indole present in the belly meat at 0, 2, or 5 hr postmortem, but a significant difference became apparent 10, 15, and 24 postmortem. The content of indole in the roe was still low, however, even 24 hr postmortem (49.17 ng/g).

S‐containing compounds, which are also associated with irritating odors, were identified at 5 hr postmortem in accordance with the sensory evaluation. These levels are closely related to s‐containing free amino acids, thiamine, and glutathione (Girard & Durance, 2000).

## 4 CONCLUSIONS

In a series of Chinese mitten crab samples, the sensory evaluation scores decreased as time postmortem increased. A noticeable odor of spoilage was present by 10 hr postmortem. The TVBN value (23.67 mg N/100 g at 24 hr postmortem) increased and then decreased as time progressed. Biogenic amines were detected at 5 hr postmortem and 24 hr postmortem, but at relatively low (non‐poisonous) levels. Trimethylamine was detected in living crab belly meat and 2 hr postmortem (8.33 µg/g at 24 hr) in crab roe. Indole was detected at 0 hr (belly meat) and 10 hr (crab roe) postmortem, but did not change significantly throughout the observation period. Sulfur‐containing compounds were detected 5 hr postmortem and gradually increased as time postmortem increased. The samples did not exceed the legal limit for TVBN within the relatively brief storage period (24 hr), but still showed relatively high levels of biogenic amines and volatile compounds. These indicators suggest that the crab was already corrupted by 10 hr postmortem. Chinese mitten crabs that have been stored postmortem overnight are thus not recommended for consumption due to their unacceptable sensory evaluation scores and high TVC content. Future study may focus on different processing methods (steaming, frying) on the qualify aspects of the crab.

## CONFLICT OF INTEREST

The authors declare that they do not have any conflict of interest. This study does not involve any patients or animal testing.
